# Effect of mindfulness-based stress on cancer-related fatigue in breast cancer patients: a systematic review and meta-analysis

**DOI:** 10.3389/fonc.2025.1585383

**Published:** 2025-09-29

**Authors:** Xiong Wang, Lei Gao, Yuqi Pang, Ting Huang

**Affiliations:** ^1^ Department of Anesthesiology Department Luzhou Maternal & Child Health Hospital (Luzhou Second People's Hospital), Luzhou, Sichuan, China; ^2^ School of Nursing, Yingtan Health Vocational College, Yingtan, Jiangxi, China; ^3^ Health Care College of Yingtan Health Vocational College, Yingtan, Jiangxi, China

**Keywords:** MBSR, breast cancer, cancer-related fatigue, meta-analysis, fatigue

## Abstract

**Background:**

Cancer-related fatigue (CRF) can seriously affect the quality of life of breast cancer patients. Mindfulness-based stress reduction therapy (MBSR) has been increasingly used in the treatment of breast cancer patients to reduce psychological distress and promote emotional and physical health. This review aims to provide an updated assessment of the role of MBSR in reducing CRF and sleep quality in breast cancer patients.

**Objective:**

To evaluate the effect of MBSR on cancer-related fatigue in patients with breast cancer.

**Methods:**

Computer search PubMed, Embase, Web of Science, Wanfang and other databases, We were able to search for articles related to CRF from MBSR in breast cancer patients from the establishment of the database to October 2023. Researchers independently screened the literature and extracted information. The meta-analysis was performed using Review Manager Software (version 5.3).

**Results:**

A meta-analysis of 11 studies included showed that MBSR could reduce CRF in breast cancer patients (SMD = -0.86, 95%CI = −1.22 ~ −0.50). Improved sleep quality (MD = -2.54, 95%CI = −3.38 ~ −1.70).

**Conclusion:**

Our research indicates that MBSR can reduce CRF and improve sleep quality in breast cancer patients. However, due to the moderate and significant heterogeneity in the quality of evidence observed in the included studies, these findings should be interpreted with caution.

## 1 Introduction

Breast cancer is the most common malignant tumour in women worldwide, posing a severe threat to women’s health and life. According to the survey on the incidence of female breast cancer in 185 countries in 2020, about 2.3 million female cases are diagnosed with milk adenocarcinoma every year, accounting for 11.7% of new cancer cases (1). However, in recent years, the emergence of neoadjuvant therapy has increased the treatment probability and improved the survival rate of patients. The global 5-year survival rate for breast cancer is currently 90% (2). Survivors are also faced with a series of physical and mental problems, such as premature menopause, body image disorder, fatigue, depression, etc (3–5). For breast cancer patients during chemotherapy, cancer-related fatigue (CRF) is the most common complication, and the incidence can be as high as 59% to 100% (6). CRF is a multidimensional concept that affects the physical (less energy and more need of sleep), cognitive (diminished concentration and attention), and affective (diminished motivation) domains (7).CRF is more severe and long-lasting than ordinary fatigue and cannot be relieved by rest and sleep. Therefore, improving CRF in breast cancer patients has been a research hotspot in recent years.

Mindfulness-Based Stress Reduction (MBSR) is an increasingly popular comprehensive psychosocial intervention, potentially through mechanisms that enhance mindfulness during meditation practice, such as sustaining moment-to-moment attention, flexibly shifting attentional focus among thoughts and sensations, and fostering non-elaborative awareness by recontextualizing negative experiences (8, 9).

Many patients with breast cancer turn to complementary therapies to deal with the symptoms of the disease (10, 11). A total of 33% to 47% of women worldwide and 48% to 80% of American women make use of such therapies, and meditation is one of complementary alternatives that positively influences the rehabilitation by reducing pain, stress, anxiety, depression, fatigue, and even the side effects caused by drug treatments (12, 13). Although meta-analysis have reported the effect of MBSR on breast cancer patients, no effect on CRF has been reported (14). Haller (15)only discussed the impact of MBSR on CRF in breast cancer patients in meta minutes, there are few pieces of literature included and a lack of reports on the effect on sleep quality. This study comprehensively collected the literature of randomized controlled studies of MBSR on CRF in breast cancer patients. The result of the intervention was verified through meta-analysis, which provided authoritative, reliable and complete evidence support for the clinical nursing work of breast cancer patients and an evidence-based basis for medical care decision-making.

## 2 Methods

This study was reported per the PRISMA NMA guidelines (16). The review protocol was registered with the International Prospective Register of Systematic Review (PROSPERO CRD420251130977).

### 2.1 Literature search

This meta-analysis followed the guidelines outlined in the systematic review and meta-analysis Preferred Reporting Program. Two independent researchers thoroughly searched multiple databases, including PubMed, Web of Science, Cochrane Library, CNKI, Wan Fang Data, and VIP. The search period was extended from the date of the establishment of the library until August 2025. Search is limited to articles published in Chinese or English. The search terms, including These searches, were performed using the following keywords: “Breast Neoplasm”, “Neoplasm, Breast,” “Breast Tumors”, “Breast Tumor”, “Tumor, Breast”, “Tumors, Breast”, “Neoplasms, Breast”, “Breast Cancer”, “Cancer, Breast”, “Mammary Cancer”, “Cancer Mammary”,”Cancers, Mammary”, “Mammary Cancers”, “Malignant Neoplasm of Breast”, “Breast Malignant Neoplasm”, “Breast Malignant Neoplasms”, “Malignant Tumor of Breast”, “Breast Malignant Tumor”, “Breast Malignant Tumors”, “Cancer of Breast”, “Cancer of the Breast”, “Fatigue”, “mindfulness-based stress reduction”, “mindfulness-based stress reduction”, “MBSR”, “mindfulness-based cognitive therapy”, “MBCT”, “mindfulness-based intervention”.

### 2.2 Inclusion and exclusion criteria

Study type: Randomized Controlled trial (RCT). The subjects were breast cancer patients with a definite pathological or cytological diagnosis.

Interventions: Patients used various types of MBSR

Result: CRF score, sleep quality

Exclusion criteria: Non-Chinese-English literature; Duplicate publications; Unable to obtain the required data; The index results must conform to the literature.

### 2.3 Data extraction

The two authors independently extracted data from all eligible studies. The following variables were removed from each study: first author name, year of publication, country, study design, sample size, age, sex ratio, and study quality score. Any disagreements are resolved through discussion or consultation with senior reviewers to reach a consensus.

### 2.4 Quality assessment

The authors independently assessed the risk of bias in included RCTS using the Cochrane assessment tool, which included the following seven areas: “adequate sequence generation, assignment concealment, participant and person blinding, outcome assessment blinding, incomplete outcome data, selective reporting, and other bias”.

### 2.5 Data synthesis and analysis

RevMan 5.3 software was used for analysis. If there was no statistical significance in the heterogeneity among the results (*P*≥ 0.1, *I^2^* **<** 50%), a fixed effect model was used for meta-analysis. If the heterogeneity among the findings was statistically significant (*P* < 0.1, *I^2^* ≥50%), the random-effects model was used for meta-analysis after the influence of apparent clinical heterogeneity was excluded. For continuous data, Weighted Mean Difference (*WMD*) was used to analyze results obtained with the same measurement tools. Standardized Mean Difference (*SMD*) was used to analyze the same variables when different measurement tools were used. 95%CI was calculated for all analyses.

## 3 Results

### 3.1 Study selection

**Figure 1:**
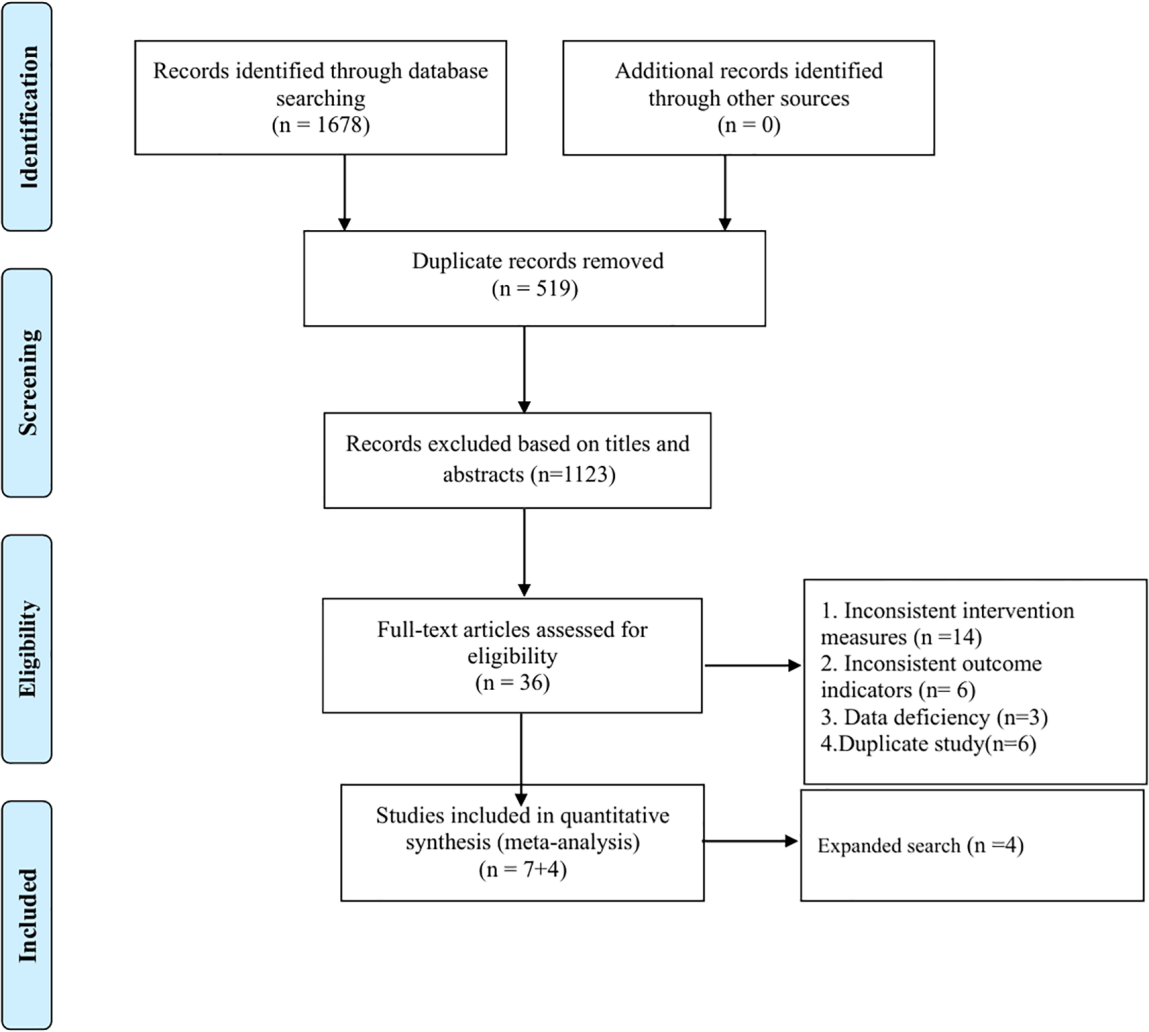
Flow chart.

The literature search yielded 1678 results, most of which were excluded because they were replicates or because they were not relevant to our meta-analysis. Then, the full text of 36 articles is reviewed. Finally, 11 literatures were included in this study. See **Figure 1**. Flow chart.

### 3.2 Basic characteristics of included studies

Our research included a total of 11 studies, among which 6 were from China, 4 from the USA, and 1 from Japan. Other characteristics are detailed in **Table 1**.

**Table 1:** The characteristics of the study and participants.

Author	Year	Country	Sample	Age	Sample size	Tumor staging	Intervention time	Frequency	Outcome
Park etal (17)	2020	Japan	MBSR/CG	53.21 ± 8.4/ 54.19 ± 9.27	35/36	0-III	8 weeks.	45min/day	BFI
Cao etal (18)	2016	China	MBSR/CG	35.45 ± 9.21/ 36.12 ± 9.67	100/100	I-III	8 weeks.	45min/day	CFS
Fan etal (19)	2021	China	MBSR/CG	46.32 ± 5.69/ 45.26 ± 5.42	90/90	I-III	5 weeks.	2 ~ 3 h/time, twice a day	PFS-R/PSQI
Wang etal (20)	2021	Chian	MBSR/CG	49.15 ± 8.39/ 51.00 ± 9.94	41/43	I-III	6 weeks.	Once/week.30 to 60 minutes/time	CFS/PSQI
Bower etal (21)	2015	USA	MBSR/CG	46.1/47.7	39/32	0-III	6 weeks.	6 times/week	FSI/PSQI
Reich etal (22)	2014	USA	MBSR/CG	58.0 ± 10.3/ 58.2 ± 9.5	17/24	0-III	6 weeks.	15–45 minutes/day	MDASI
Lengacher etal (23)	2015	USA	MBSR/CG	56.1 ± 9.1/ 58.0 ± 10.2	38/41	0-III	6 weeks.	6 times/week	PSQI
Jiang etal (24)	2019	China	MBSR/CG	43.48 ± 9.72/ 42.63 ± 9.56	58/50	I-III	4 weeks.	2 times/day20min/time	PSQI
Wang etal (25)	2017	China	MBSR/CG	40.67 ± 4.58/ 41.35 ± 3.59	45/46	I-IV	8 weeks	15-45min/time	PFS-R/PSQI
Cecile etal (26)	2025	USA	MBSR/CG	56.3 ± 11.4/59.5 ± 11.0	91/31	NA	6weeks	15–45 min/6time/week	PROMIS-F
Fan etal (27)	2024	China	MBSR/CG	18-78	40/40	NA	8 weeks	Once a week/40 min/time	CFS

BFI, Brief fatigue inventory; CFS, Cancer fatigue scale; FSI, Fatigue symptom inventory; PFS, The Revised Piper Fatigue Scale; MFSI-SF, multidimensional fatigue symptom inventory-short form; PSQI, Pittsburgh sleep quality index; MDASI, MD Anderson Symptom Inventory; PROMISÒ, Patient-Reported Outcomes Measurement Information System.

### 3.3 Methodological quality assessment

**Figure 2:**
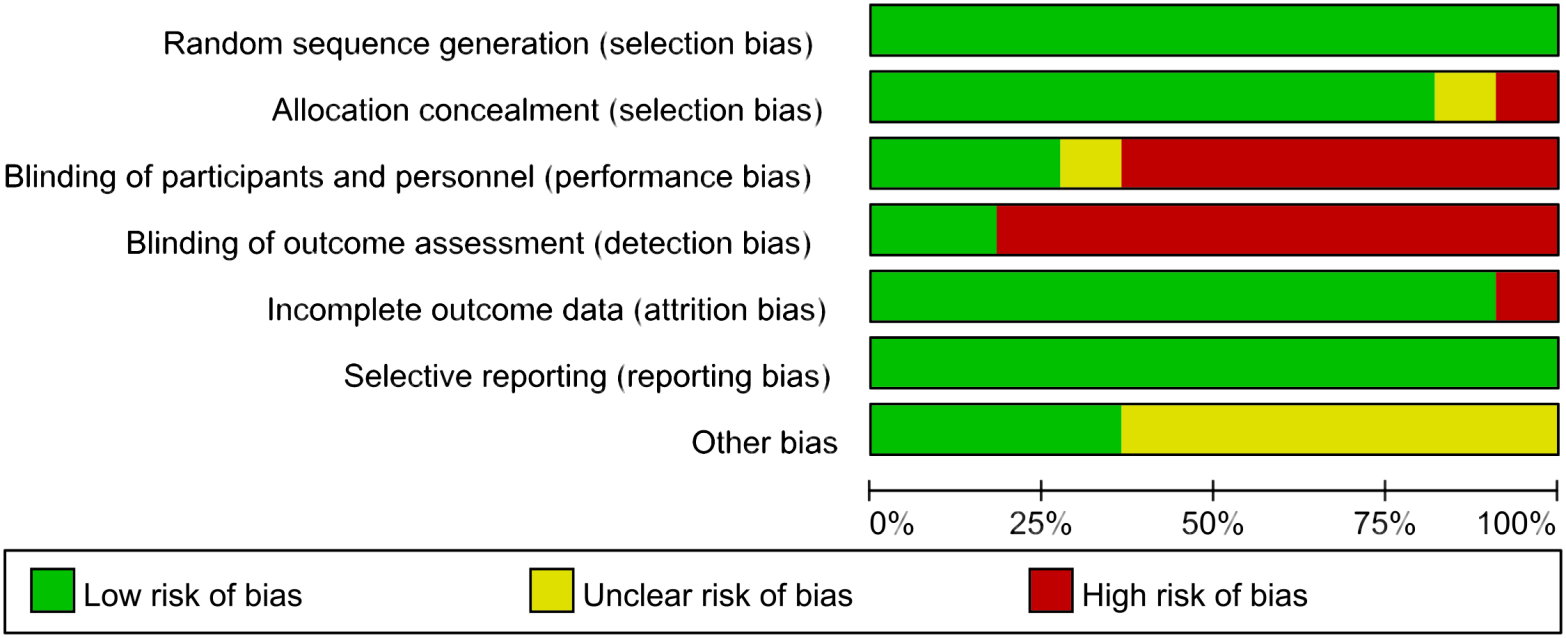
Summary of risk of bias.

The methodological quality of the included 11 articles was evaluated. The summary of the risk of bias assessment is shown in **Figure 2**.

### 3.4 Results of the meta-analysis

#### 3.4.1 CRF

**Figure 3:**
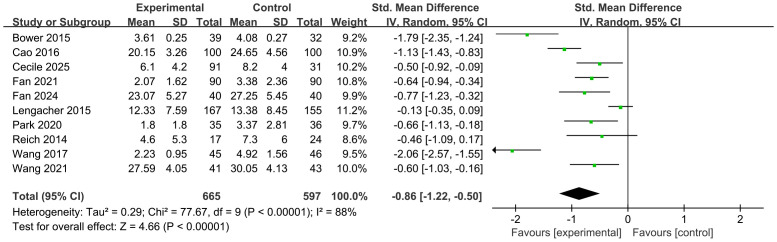
Impact of MBSR on CRF.

The efficacy of MBSR was compared with that of the control group. A meta-analysis of 10 studies was conducted, and the overall results are presented in **Figure 3**. Compared to the control group, MBSR significantly alleviated CRF [SMD = –0.86, 95% CI (–1.22, –0.50), p < 0.001], with significant heterogeneity observed (*I²* = 88%, p < 0.001). Sensitivity analysis was performed by sequentially excluding each study, yet substantial heterogeneity persisted. Therefore, subgroup analysis was conducted to explore potential sources of heterogeneity; detailed results are provided in **Table 2** and the Supplementary Materials.

**Table 2:** Subgroup analysis to assess the effect of MBSI on CRF.

Variable	Number of trials	Sample size		Meta-analysis		Heterogeneity		
		Experimental	Control	SMD	CI	I^2^	Chi^2^	P^b^
Year of publication								
Before 2020	5	368	357	-1.10	-1.86, -0.35	95%	76.28	<0.001
After 2020	5	297	240	-0.63	-0.81, -0.45	0	0.78	0.94
Country of publication								
China	5	316	319	-1.02	-1.47, -0.58	85%	26.53	<0.001
USA	4	314	242	-0.70	-1.36, -0.03	90%	30.09	<0.001
Other	1	35	36	-0.66	-1.13, -0.18	–		
Outcome measurement								
FSI	2	206	187	-0.94	-2.57, 0.69	97%	29.63	<0.001
CFS	3	181	183	-0.87	-1.20, -0.53	55%	4.43	0.11
Other	3	143	91	-0.55	-0.83, -0.27	0	0.31	0.86
PFS-R	2	135	136	-1.34	-1.22, 0.05	95%	21.84	<0.001
Intervention duration								
6 Weeks	5	355	285	-0.67	-1.19, -0.15	87%	30.83	<0.001
8 Weeks	4	220	222	-1.15	-1.68, -0.61	84%	18.79	<0.001
Other	1	90	90	-0.64	-0.94, -0.34	–		
Intervention frequency								
Once a week	2	81	83	-0.68	-1.00, -0.37	0	0.30	<0.001
6 times/week	3	297	218	-0.78	-1.63, 0.08	93%	30.03	<0.001
Other	5	287	296	-0.99	-1.47, -0.51	85%	27.16	<0.001

#### 3.4.2 PSQI

**Figure 4:**
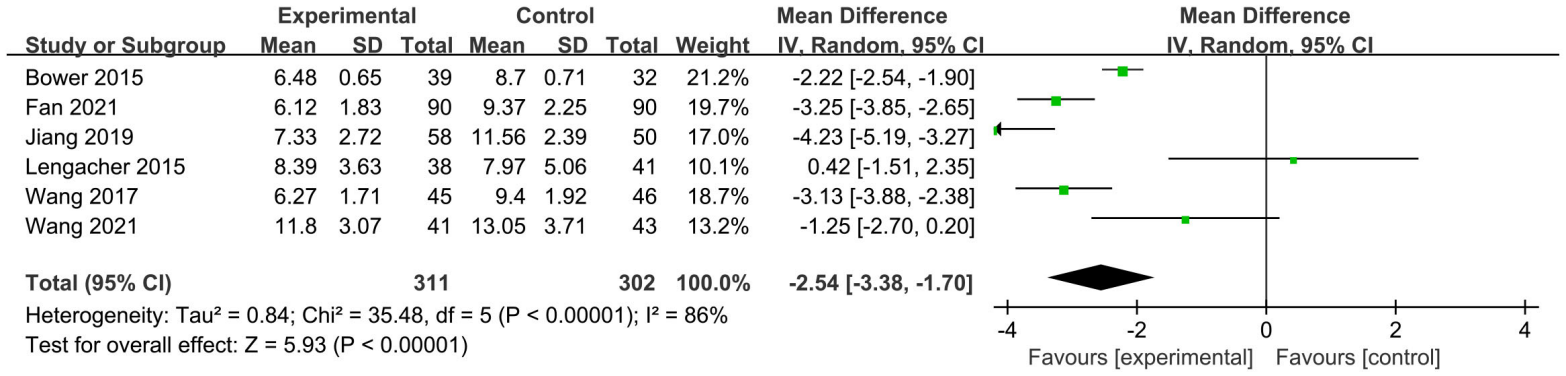
Impact of MBSR on PSQI.

The efficacy of MBSR was compared with that of a control group. A meta-analysis of six studies was performed, and the overall findings are illustrated in **Figure 4**. Compared with the control group, MBSR resulted in a significant improvement in sleep quality among breast cancer patients [MD = –2.54, 95% CI (–3.38, –1.70), p < 0.001], with significant heterogeneity detected (*I²* = 86%, p < 0.001). Sensitivity analysis conducted by sequentially excluding individual studies showed that substantial heterogeneity remained. Therefore, subgroup analyses were carried out to explore potential sources of heterogeneity; detailed results are presented in **Table 3** and the Supplementary Materials.

**Table 3:** Subgroup analysis to assess the effect of MBSR on PSQI.

Variable	Number of trials	Sample size		Meta-analysis		Heterogeneity		
		Experimental	Control	MD	CI	I^2^	Chi^2^	P^b^
Year of publication								
Before 2020	3	122	119	-2.04	-3.20, -0.88	84%	12.61	0.002
After 2020	3	189	183	-3.03	-4.35, -1.72	82%	11.23	0.004
Country of publication								
China	4	234	229	-3.11	-3.96, -2.26	74%	11.34	0.01
Other	2	311	73	-1.08	-3.64, 1.48	86%	6.98	0.008
Intervention duration								
6 Weeks	3	118	116	-1.26	-2.70, 0.17	76%	8.39	0.02
Other	3	193	186	-3.45	-4.03, -2.87	44%	3.59	0.17

#### 3.4.3 Publication bias

**Figure 5:**
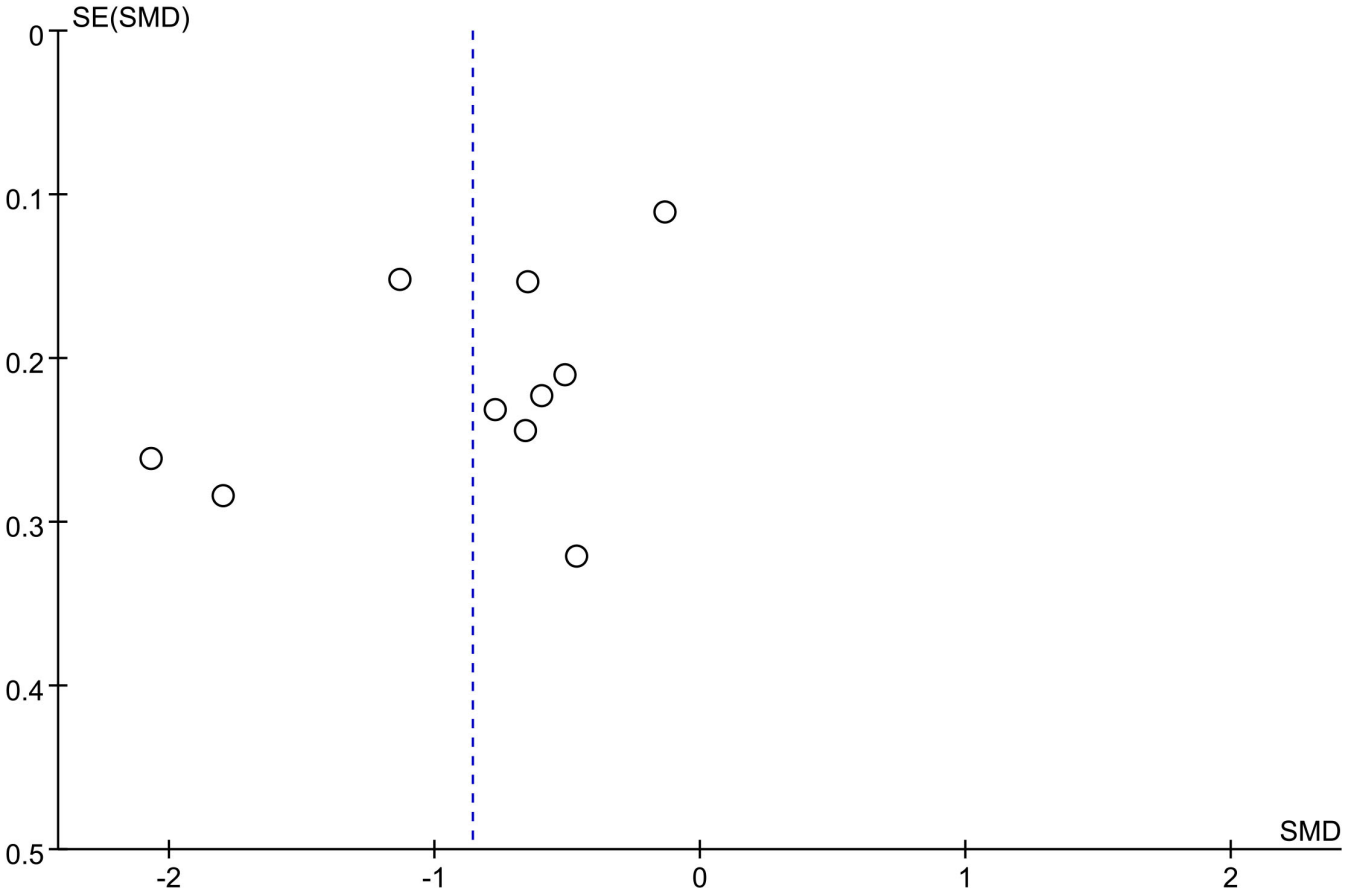
Funnel plot.

The funnel plot showed potential publication bias (**Figure 5**). The slight asymmetry observed in the funnel plot suggests the possible presence of publication bias among the included studies.

## 4 Discussions

CRF not only restricts the normal physiological function of breast cancer patients but also harms the mental health of patients, seriously reduces the quality of life of patients, and ultimately affects the progress of cancer treatment. This study found that the use of MBSR in women with breast cancer was associated with improved sleep quality in breast cancer patients by reducing CRF.

Xie (28) discussed the impact of MBSR on CRF in cancer patients in her study. results found that MBSR could reduce CRF in cancer patients, but the effects of MBSR on CRF were not apparent. In our study, MBSR reduced CRF in breast cancer patients. Our findings are identical to those of the Castanhel (29) study. The neural mechanism of MBSR is that long-term mindfulness training can enhance the activity of theta waves in the frontal lobe of the brain, stimulate the activity levels of related brain regions such as attention and emotion, and increase the gray matter density in regions such as the left superior temporal gyrus and the right hippocampus (30, 31). Furthermore, Research has found that when breast cancer patients undergo MBSR training, the interleukin-4 (IL-4), a product of T cells, increases in their bodies. This cytokine plays a crucial regulatory role in the differentiation and maturation of immune cells, which may be the neuroimmunological basis for enhancing the body’s immunity through MBSR (32). The psychological mechanism by which MBSR improves CRF may involve positive changes in an individual’s awareness and attention towards things and related experiences, enhancing the response to external stimuli, strengthening self-regulation functions, reducing fatigue, and promoting physical and mental health (33). CRF can trigger a decline in physical function in breast cancer patients (34)and is a significant problem during breast cancer treatment (35). Armes et al. (36)emphasize that no medication for fatigue exists. However, interventions should focus on psychological, educational, social, and group support therapies designed to allow individuals to explain fatigue, cope with symptoms with positive thinking, and return to daily activities. The main components of MBSR include sitting and walking meditation, yoga practice, and mindfulness relaxation techniques. MBSR can improve patients’ cortisol and blood pressure, reduce the subjective pain and immune response caused by stress, and enhance and activate the structure of brain regions involved in emotional processing (37). Thus, it is beneficial to improve patients’ CRF.

Few studies have focused on the effect of MBSR on sleep quality in breast cancer patients. Good sleep benefits the human spirit, emotion, immune function, cell growth and repair. The disturbance of sleep patterns and the decline of sleep quality will affect patients’ cognitive function, immune function and quality of life. Breast cancer patients often worry about their disease, and their sleep quality decreases. In addition, chemotherapy causes hair loss, gastrointestinal reactions, bone marrow suppression, anxiety and depression, and other problems that aggravate patients’ sleep disorders. Breast cancer patients have a higher incidence of sleep disorders during chemotherapy, up to 80% (38). MBSR is a mindfulness-based stress management therapy that aims to pass mindfulness-based meditation. The self-management mode of training can reduce the pressure on the subject and effectively manage the subject’s emotions (39). When MBSR is applied to breast cancer patients, it can regulate the quality of sleep by relieving their mental state of anxiety and depression and improving their emotional regulation ability.

## 5 Limitations and future implications

This study has several limitations. Only literature published in Chinese and English was included, and the search was limited to a finite number of databases, which may have resulted in an incomplete retrieval of relevant studies. Variations in intervention protocols, session durations, and outcome measures across the included studies may have introduced heterogeneity and potential bias. The long-term effects of mindfulness-based interventions on CRF and sleep quality in breast cancer patients remain to be further investigated. Additionally, the relatively small number of included studies, coupled with insufficient reporting of blinding and allocation concealment procedures, may compromise the methodological quality and limit the robustness of the conclusions. Future research should involve more rigorous, high-quality randomized controlled trials with larger sample sizes to further evaluate the efficacy of mindfulness-based stress reduction in improving CRF and sleep outcomes in this population.

## 6 Conclusion

Our combined findings suggest that MBSR reduces CRF and improves sleep quality in breast cancer patients. Given some limitations of this meta-analysis, well-designed studies with larger sample sizes and more detailed outcome measures are needed.

## Data Availability

The original contributions presented in the study are included in the article/supplementary material. Further inquiries can be directed to the corresponding author.
